# Interaction of chorioamnionitis at term with maternal, fetal and obstetrical factors as predictors of neonatal mortality: a population-based cohort study

**DOI:** 10.1186/s12884-020-03142-0

**Published:** 2020-08-08

**Authors:** Dina Zaki, Jaques Balayla, Marc Beltempo, Guillaume Gazil, Anne Monique Nuyt, Isabelle Boucoiran

**Affiliations:** 1 Department of Obstetrics and Gynecology, Faculty of Medicine, Université de Montréal, 3175 Côte Sainte-Catherine, Montreal, QC H3T 1C5 Canada; 2 Department of Obstetrics and Gynecology, McGill University Health Centre (MUHC), McGill University, Montreal, Canada; 3 Department of Pediatrics, Montreal Children’s Hospital, McGill University Health Centre, Montreal, QC Canada; 4 Applied Clinical Research Unit (URCA), Centre de recherche du CHU Sainte-Justine, Montreal, QC Canada; 5 Department of Pediatrics, Faculty of Medicine, Université de Montréal, Montreal, QC Canada; 6 CHU Sainte-Justine Research Centre, Montreal, QC Canada; 7 Department of Social and Preventive Medicine, Faculty of Medicine, Université de Montréal, Montreal, QC Canada

**Keywords:** Neonatal death, Chorioamnionitis, Smoking, Early term

## Abstract

**Background:**

Chorioamnionitis is a frequent complication of pregnancy and is known to be associated with serious adverse post-natal outcomes including death. However, the assessment of fetal well-being in labor in the context of chorioamnionitis is often challenging because of fetal tachycardia. Identifying specific risk factors for adverse neonatal outcomes in the context of chorioamnionitis could therefore be of paramount importance. This study aimed to determine if maternal and fetal risk factors for increased neonatal mortality and early neonatal mortality are modified in the context of chorioamnionitis in term pregnancies.

**Methods:**

A retrospective population-based cohort study using the United States birth/infant death public file from 2011 to 2013 was performed, including all live births at 37 weeks gestation and beyond. Interaction between chorioamnionitis and maternal demographic variables as well as labor and delivery potential risk factors were analyzed for association with neonatal death (< 28 days) and early neonatal death (< 7 days) using multivariate logistic regressions.

**Results:**

Among 9,034,428 live births, the prevalence of chorioamionitis was 1.29% (95% CI 1.28–1.30%). The incidence of neonatal death and early neonatal death were 0.09 and 0.06% in the chorioamnionitis group versus 0.06 and 0.04% in the no chorioamnionitis group (*p* = 0.0003 and < 0.0001), respectively. Smoking was significantly associated with neonatal death and early neonatal death in the context of chorioamnionitis (OR 2.44, CI:1.34–4.43/ 2.36 CI:1.11–5.01) but was either less strongly or not associated in the absence of chorioamnionitis (OR 1.24, CI:1.14–1.35/0.93, CI:0.82–1.05). The association between gestational age (37 weeks compared to 39 weeks) and neonatal death was more important in the context of chorioamnionitis (OR = 3.19, CI: 1.75–5.82 versus 1.63, CI: 1.49–1.79). Multivariate analysis identified the following risk factors for neonatal death and/or early neonatal death: low maternal education, extreme maternal age, obesity (BMI > 35 kg/m^2^), late or no prenatal care, diabetes, meconium-stained amniotic fluid, gestational ages other than 39 weeks, neonatal weight < 2500 g and delivery by vacuum or caesarian.

**Conclusions:**

Smoking as well as early term have a positive interaction with chorioamnionitis for the risk of neonatal mortality. This should be taken into account when counseling pregnant women and managing laboring pregnant women with suspected chorioamnionitis.

## Background

Chorioamnionitis is a frequent complication of pregnancy and is known to be associated with serious maternal, fetal and long term-postnatal adverse outcomes including stillbirth, neonatal sepsis, chronic lung and brain neonatal diseases including hypoxic ischemic encephalopathy leading to long-term disabilities as well as maternal post-partum infections and sepsis [1].

Term delivery is associated with a lower complication rate than preterm delivery, yet the impact of chorioamnionitis in this population is less studied. Furthermore, the majority of term infants have less follow-up than their preterm counterparts however chorioamnionitis could be a risk factor requiring additional follow-up.

Chorioamnionitis is most often due to ascending bacteria causing acute inflammation of the membranes and the chorion of the placenta. Risk factors for chorioamnionitis include longer duration of membrane rupture (more than 12 h), prolonged labor, nulliparity, internal monitoring of labor, multiple vaginal exams (more than or equal to 3), meconium-stained amniotic fluid, smoking, alcohol or drug abuse, immune compromised states and colonization with group B streptococcus. (1) The importance of clinical diagnosis in the pregnant woman has been underlined by its association with fetal and maternal mortality if left untreated [2]. Studies have shown that chorioamnionitis was associated with an approximately 2- and 3.5- fold increased odds of neonatal adverse outcomes < 34 and > 34 weeks respectively regardless of chorioamnionitis duration [3].

However, to our knowledge, no study has examined the effect specific maternal, fetal and obstetrical risk factors on neonatal mortality in the context of chorioamnionitis in term infants. Recognizing these risk factors could be an asset when tailoring protocols for the clinical management of chorioamnionitis. This study aimed to determine if maternal and fetal risk factors for increased neonatal mortality and early neonatal mortality are modified in the context of chorioamnionitis at term.

## Methods

We performed a retrospective, population-based cohort study using the US Centers for Disease Control and Prevention (CDC) 2011–2013 linked birth/infant death public data set. This data is used for monitoring and exploring relationships between infant death and risk factors present at birth. It contains information on approximately 4 million annual live births in the United States (U.S.), and includes a number of obstetrical, perinatal and infant characteristics. The information from the death certificate is linked to information from the birth certificate for each infant under 1 year of age. This allows the US CDC to use many additional variables available from the birth certificate to conduct analyses of infant mortality patterns. This file is publicly available and does not contain any identification data (https://www.cdc.gov/nchs/nvss/linked-birth.htm).

All live births at 37 weeks or more were included. Multiple gestations and fetuses with severe congenital anomalies were excluded. These were determined using the CDC dictionary of congenital anomalies and include anencephaly, congenital hydrocephalus, spina bifida, other congenital malformations of the nervous, circulatory, respiratory, digestive, genitourinary and musculoskeletal systems.

The outcomes measured were (1) neonatal death defined as death in the first 28 days after live birth and (2) early neonatal death defined as infant death in first 7 days of life.

Chorioamnionitis was reported if documented on the birth record as “clinical chorioamnionitis”. Detailed clinical criteria for the diagnosis of chorioamnionitis were not available. Other potential risk factors for neonatal death were identified based on literature review and availability in the US CDC public files. These maternal and fetal variables are outlined in Table 1.

**Table 1:** Demographics and Characteristics of Mothers and Babies Stratified by Chorioamnionitis - n%

Characteristic	Total (***N*** = 9,034,428)	Chorioamnionitis (***N*** = 116,627)	No chorioamnionitis(***N*** = 8,917,801)	***p***-Value	Unknown
**Neonatal Death**	5794 (0.1)	106 (0.1)	5688 (0.1)	0.0003	
**Early Death**	3391 (0.0)	71 (0.1)	3320 (0.0)	< 0.001	
**Late Death**	2403 (0.0)	35 (0.0)	2368 (0.0)	0.4720	
**Admission to NICU**	339,942 (3.8)	29,488 (25.3)	310,454 (3.5)	<.0001	5414 (0.1)
**Mother’s Age Category (years)**				<.0001	
< 20	691,055 (7.6)	13,310 (11.4)	677,745 (7.6)		
20–24	2,096,978 (23.2)	31,111 (26.7)	2,065,867 (23.2)		
25–29	2,614,045 (28.9)	33,249 (28.5)	2,580,796 (28.9)		
30–34	2,328,649 (25.8)	26,609 (22.8)	2,302,040 (25.8)		
35–39	1,056,573 (11.7)	10,137 (8.7)	1,046,436 (11.7)		
> = 40	247,128 (2.7)	2211 (1.9)	244,917 (2.7)		
**Pre-pregnancy Body Mass Index (kg/m**^**2**^**)**				<.0001	36,428 (4)
Underweight < 18.5	330,184 (3.7)	4023 (3.4)	326,161 (3.7)		
Normal 18.5–24.9	4,101,894 (45.4)	57,298 (49.1)	4,044,596 (45.4)		
Overweight 25.0–29.9	2,209,657 (24.5)	29,045 (24.9)	2,180,612 (24.5)		
Obesity I 30.0–34.9	1,141,225 (12.6)	13,188 (11.3)	1,128,037 (12.6)		
Obesity II 35.0–39.9	530,684 (5.9)	5453 (4.7)	525,231 (5.9)		
Extreme Obesity III > =40	356,516 (3.9)	3263 (2.8)	353,253 (4.0)		
**Mother’s Education**				<.0001	101,440 (1.1)
Primary / High School	1,489,421 (16.5)	16,434 (14.1)	1,472,987 (16.5)		
Undergraduate	4,108,603 (45.5)	52,287 (44.8)	4,056,316 (45.5)		
Academic Degree	2,392,987 (26.5)	32,652 (28.0)	2,360,335 (26.5)		
Doctorate or Professional Degree	941,977 (10.4)	13,867 (11.9)	928,110 (10.4)		
**Marital Status**	5,436,538 (60.2)	64,979 (55.7)	5,371,559 (60.2)	<.0001	
**Mother’s Race**				<.0001	
White	6,988,814 (77.4)	83,482 (71.6)	6,905,332 (77.4)		
Black	1,353,358 (15.0)	17,532 (15.0)	1,335,826 (15.0)		
American Indian / Alaskan Native	93,982 (1.0)	1058 (0.9)	92,924 (1.0)		
Asian / Pacific Islander	598,274 (6.6)	14,555 (12.5)	583,719 (6.5)		
**Resident Status**	6,627,740 (73.4)	87,787 (75.3)	6,539,953 (73.3)	<.0001	
**Nulliparous**	3,639,997 (40.3)	94,060 (80.7)	3,545,937 (39.8)	<.0001	389,941 (4.3)
**Smoker**	718,229 (7.9)	5415 (4.6)	712,814 (8.0)	< 0.001	511,070 (5.7)
**Chronic Hypertension**	112,002 (1.2)	1440 (1.2)	110,562 (1.2)	0.8660	9224 (0.1)
**Pregnancy Associated Hypertension**	349,609 (3.9)	6802 (5.8)	342,807 (3.8)	<.0001	9224 (0.1)
**Diabetes (overall)**	494,901 (5.5)	7103 (6.1)	487,798 (5.5)	<.0001	9224 (0.1)
Pre-pregnancy Diabetes	57,013 (0.6)	685 (0.6)	56,328 (0.6)	0.0566	9224 (0.1)
Gestational Diabetes	437,888 (4.8)	6418 (5.5)	431,470 (4.8)	<.0001	9224 (0.1)
**Month Prenatal Care Began**				<.0001	300,509 (3.3)
1st to 3rd month	6,474,810 (71.7)	85,726 (73.5)	6,389,084 (71.6)		
4th to 6th month	1,744,616 (19.3)	21,724 (18.6)	1,722,892 (19.3)		
7th to final month	408,509 (4.5)	5174 (4.4)	403,335 (4.5)		
No prenatal care	105,984 (1.2)	948 (0.8)	105,036 (1.2)		
**Meconium-stained amniotic fluid**	491,605 (5.4)	20,313 (17.4)	471,292 (5.3)	<.0001	
**Tocolysis**	55,479 (0.6)	1281 (1.1)	54,198 (0.6)	<.0001	13,226 (0.1)
**Induction of Labor**	2,221,042 (24.6)	37,531 (32.2)	2,183,511 (24.5)	<.0001	
**Final Route and Method of Delivery**				<.0001	4106 (0.0)
Spontaneous	5,976,036 (66.1)	61,161 (52.4)	5,914,875 (66.3)		
Forceps	58,786 (0.7)	2529 (2.2)	56,257 (0.6)		
Vacuum	270,410 (3.0)	8732 (7.5)	261,678 (2.9)		
Cesarean	2,725,090 (30.2)	44,186 (37.9)	2,680,904 (30.1)		
**Gestational Age (weeks)**				<.0001	
37	825,690 (9.1)	7647 (6.6)	818,043 (9.2)		
38	1,667,050 (18.5)	16,174 (13.9)	1,650,876 (18.5)		
39	3,078,494 (34.1)	32,304 (27.7)	3,046,190 (34.2)		
40	2,025,919 (22.4)	33,690 (28.9)	1,992,229 (22.3)		
41 and over	1,437,275 (15.9)	26,812 (23.0)	1,410,463 (15.8)		
**Birth Weight Category (g)**				<.0001	
< 2500	237,523 (2.6)	1740 (1.5)	235,783 (2.6)		
2500–3999	7,994,850 (88.5)	101,877 (87.4)	7,892,973 (88.5)		
> 4000	802,055 (8.9)	13,010 (11.2)	789,045 (8.8)		
**Sex of Infant**				<.0001	
Female	4,434,570 (49.1)	55,583 (47.7)	4,378,987 (49.1)		
Male	4,599,858 (50.9)	61,044 (52.3)	4,538,814 (50.9)		

### Statistical analysis

Descriptive statistics were computed for all variables of interest. Results were grouped by chorioamnionitis status for each outcome (early death and total neonatal death). Odds ratios by chorioamnionitis status were also computed between each outcome and risk factor from the same model to explore their association and their interaction with chorioamnionitis. In addition, the potential effect of chorioamnionitis (and its importance) on these associations was evaluated graphically using interaction plots.

For modelling each outcome, intermediate multivariable logistic models were used with chorioamnionitis and each covariate as independent variables, as well as the interaction between them. To evaluate which variables would be a candidate for each final model, *p*-values from the aforementioned modelling were taken into account. Variables and interaction factors with a p-value lower than 0.2 were considered for the next step. Any variable with an odds ratio (OR) lower than 1.3 was deemed to have a weak association with the outcome and was therefore discarded for the multivariate analysis. In addition, interactions with strong to moderate importance were the only ones kept for the multivariate analysis in each final model (change in any parameter estimate greater than 20%).

For the multivariate analysis, similar variables were grouped together to build intermediate models. Variables were dropped if the *p*-value became higher than 0.05. Finally, variables from the intermediate models were sequentially combined together, and those with a p-value smaller than 0.05 were kept to obtain the final model. 95% confidence intervals (CI) were computer for adjusted OR. This analysis included only women with complete data.

All of the analyses were conducted using SAS 9.4 software (SAS Institute Inc. Cary, NC, USA).

This study was approved by the CHU Sainte-Justine ethics committee.

## Results

**Fig. 1:**
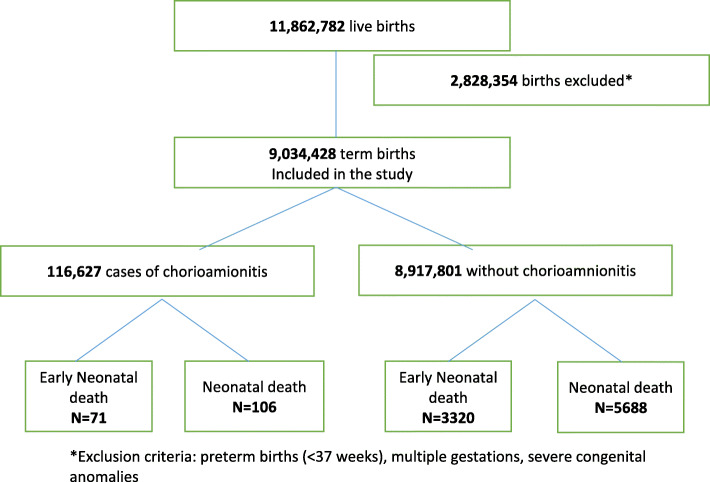
Study flowchart. *Exclusion criteria: preterm births (< 37 weeks), multiple gestations, severe congenital anomalies

Among 11,862,782 live births, 9,034,428 were included, among which 116,627 (1.29%) had documented chorioamionitis (95% confidence interval (CI): 1.28–1.30%, Fig. 1). Details on demographics and risk factors by chorioamnionitis status are presented in Table 1. The number of neonatal deaths and early neonatal deaths were respectively 106 (0.09%) and 71 (0.06%) in the chorioamnionitis group versus 5688 (0.06%) and 3320 (0.04%) in the no chorioamnionitis group (*p* = 0.0003 and < 0.0001). The crude odds ratio for the association between chorioamnionitis and neonatal mortality was 1.42 (95% CI: 1.18–1.73).

**Fig. 2:**
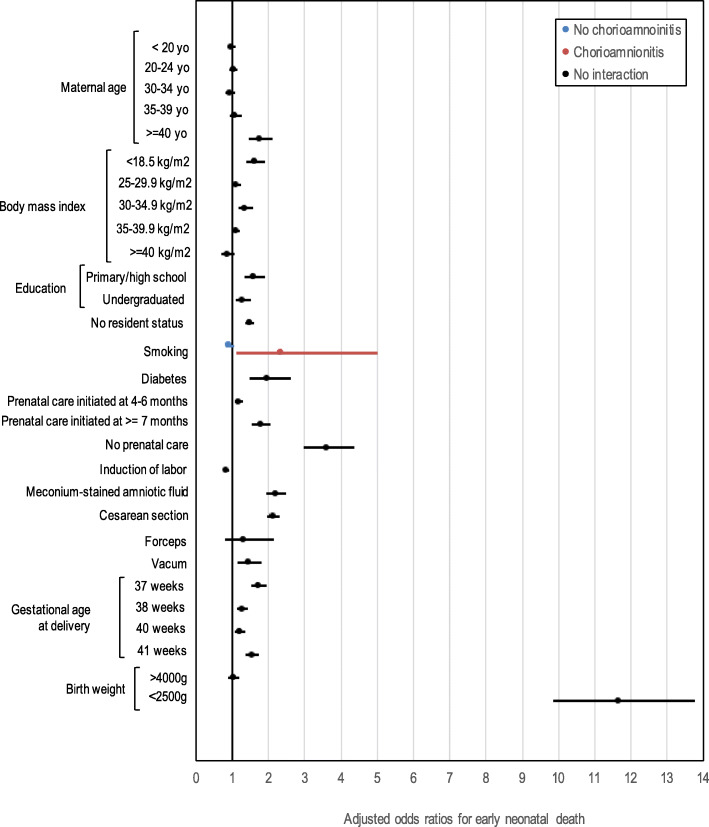
Forest plot, Adjusted odds Ratios for Early neonatal death. Birth weight reference group: 2500-2999 g. Gestational age reference group: 39 weeks. Prenatal care reference group: 1st-3rd month of gestation. Maternal age reference group: 25–29 years old. BMI reference group: 18–24.9 kg/m^2^. Education reference group: doctorate/professional degree

**Fig. 3:**
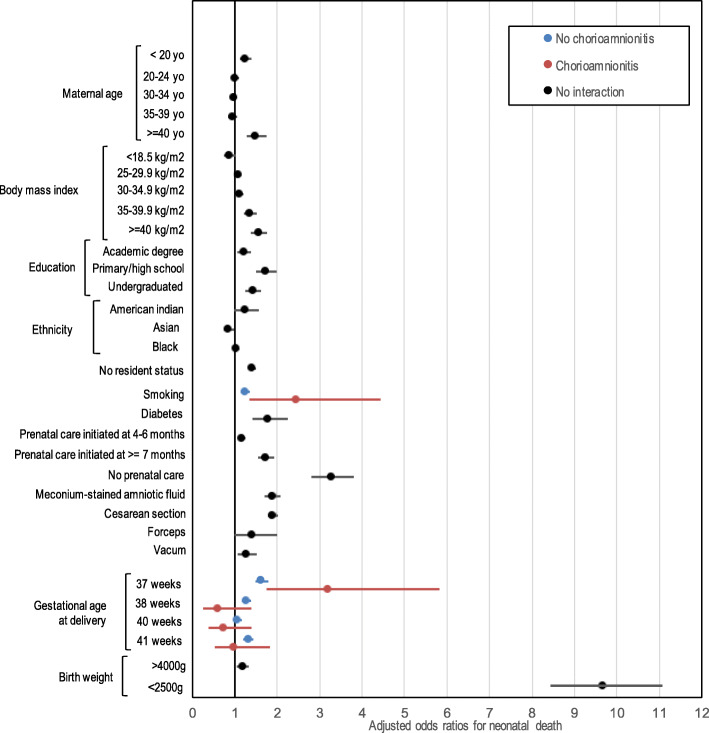
Forest plot, Adjusted odds ratios for neonatal death. Gestational age reference group: 39 weeks. Birth weight reference group: 2500-2999 g. Prenatal care reference group: 1st-3rd month of gestation. Maternal age reference group: 25–29 years old. BMI reference group: 18–24.9 kg/m^2^. Education reference group: doctorate/professional degree. Ethnicity reference group: white

The odds ratios from the final models are presented in Figs. 2 and 3. Smoking was significantly associated with *early* neonatal death in the context of chorioamnionitis (OR 2.36, 95% CI: 1.11–5.01) but was not associated in the absence of chorioamnionitis (OR 0.93, 95% CI:0.82–1.05). For *neonatal death*, the impact of gestational age (37 weeks compared to 39 weeks) was more important in the context of chorioamnionitis (OR = 3.19, 95% CI: 1.75–5.82 versus 1.63, 95% CI: 1.49–1.79). Same wise, the impact of smoking on the risk of neonatal death was higher in the context of chorioamnionitis (OR 2.44, 95% CI:1.34–4.43 versus 1.24, 95% CI:1.14–1.35).

The main risk factors for *early* neonatal death were maternal age over 40 (OR 1.75. 95% CI:1.45–2.11), obesity (OR 1.62, 95% CI:1.38–1.90), late or no prenatal care (OR 3.6, 95% CI:2.97–4.39), all gestational ages other than 39 weeks (37, 38, 40 and 41, (OR 1.7, 1.2, 1.2 and 1.53 respectively), delivery by c-section (OR 2.12, 95% CI:1.96–2.30), vacuum (OR 1.43, 95% CI: 1.14–1.80), meconium-stained amniotic fluid (OR 2.19, 95% CI: 1.93–2.48) and birth weight < 2500 g (OR 11.64, 95% CI:9.85–13.76). Finally, Induction of labor was associated with a decreased risk of early neonatal death with an OR of 0.82 (95% CI 0.75–0.91).

The main risk factors for *neonatal death* were extremes of maternal age: < 20 and > 40 (OR 1.24, 95% CI:1.11–1.39 and 1.49, 95% CI: 1.28–1.74 respectively), late or no prenatal care (OR 3.26, 95% CI: 2.80–3.80), diabetes (OR 1.78, 95% CI:1.41–2.24), meconium-stained amniotic fluid (OR 1.87, 95% CI: 1.7–2.07), delivery by c-section (OR 1.89, 95% CI: 1.78–2.01), vaccum (OR 1.27, 95% CI: 1.06–1.51) and birth weight < 2500 g (OR = 9.66, 95% CI: 8.43–11.06).

## Discussion

In this population based retrospective cohort, a significant positive interaction was found between gestational age and smoking on the one hand and chorioamnionitis on the other hand as risk factors for neonatal death and early neonatal death, controlling for other risk factors.

The increased risk of neonatal morbidity and death associated with chorioamnionitis has previously been reported for term deliveries [4, 5]. Consistently, the severity of the maternal and more importantly the fetal inflammatory response has been associated with increased neonatal mortality and morbidity [6]. In a study realized on 2008 CDC public files, the adjusted OR of chorioamnionitis for neonatal death was 1.72, 95% CI 1.20–2.45, but the interaction between chorioamnionitis and other risk factors for neonatal death were not tested [4].

Smoking has previously been reported as a risk factor for perinatal mortality [7]. The interaction of smoking with chorioamnionitis controlling for birth weight that our study has revealed could be caused by the impact of tobacco exposure on the neonatal ability to overcome infection and inflammation that is associated with chorioamnionitis. Indeed, immunity is decreased in children of mothers who smoked during pregnancy as early as in the neonatal period [8–10].

Also, it is of note that in our study, smokers had a lower risk of developing chorioamnionitis. The decrease in immune response induced by smoking could lead to differ the onset of clinical symptoms of chorioamnionitis, which could explain a lower frequency of clinically diagnosed chorioamnionitis and subsequently less treatment and more adverse outcomes in newborns.

Early term deliveries have been previously demonstrated to be associated with worse neonatal outcomes than full term deliveries (≥39 weeks) [11]. Similarly, the association between post-term deliveries and perinatal death has been established in Swedish, English and US population based cohort studies [7, 12–15] as well as in a systematic review [15]. The positive interaction between chorioamnionitis and gestational age, found in our study even if preterm deliveries were excluded, could be explained by increased lung and brain susceptibility at gestational age less than 39 weeks.

The lack of interaction between meconium-stained amniotic fluid and chorioamnionitis is surprising as chorioamnionitis has been reported as a risk factor for meconium aspiration syndrome [5]. Other significant risk factors identified in this study were consistent with previous publications. The association of adverse perinatal outcomes with late prenatal care and lower maternal education have been reported in earlier analyses of the CDC public files [16] and in other settings [17]. Similarly, severe obesity has been consistently associated with adverse perinatal outcomes [18–20].

Lastly, induction of labor was associated with a significant decrease in neonatal mortality in our study, like it has been inconsistently reported in observational studies [21] as well as in randomized control trials [22, 23]. This association should be considered with caution as the indication for labor induction could not be taken into account in our analyses. The vacuum or cesarean delivery-increased risk of early mortality was likely not causal as obstetrical intervention is expected in cases of suspected fetal asphyxia.

The main strength of our study is that it is a population-based analysis with large number outcomes. It is the first study aimed at exploring maternal and fetal risk factors at term for neonatal death and their interaction with chorioamnionitis. Limitations of this study should be noted. First, it is a retrospective study therefore causality cannot be assumed. For this reason, prospective observational studies should be conducted. Moreover, our results are based on 2010–2013 data, because these were the years with the most complete infant mortality information that were publicly available. However, the criteria for the clinical diagnosis of chorioamnionitis have been reviewed since [24]. Furthermore, as we were using a public bank, the details on chorioamnionitis diagnostic criteria, method used for gestational age determination, treatment of chorioamnionitis and neonatal management were not available. The cause of death was also not accessible, which could have provided additional information on associations identified in the study. The proportion of women with missing data which were not included in the multivariate analysis is approximately 15%. Although this may seem elevated, it is not believed to be a limitation as it is not due to recall or selection bias-and there was no difference in the amount of missing data between the two groups. Lastly, the analyses did not include intra-uterine fetal demise which may have falsely diminished the incidence and impact of chorioamnionitis.

## Conclusions

Smoking as well as early term have a positive interaction with chorioamnionitis for the risk of neonatal mortality controlling for other risk factors. This should be taken into account when counseling pregnant women. Smoking cessation is an important adjunct to routine obstetrical care and the risks associated should be discussed during prenatal visits. It should also be considered when managing laboring pregnant women with diagnosis of chorioamnionitis, particularly if they are early term. Preventative measures to minimize development of chorioamnionitis during labor could include less vaginal exams and aiming for shorter labor. Neonatology should also be made aware of the presence of these risk factors and their known association with neonatal mortality in babies born to a mother with chorioamnionitis.

## Data Availability

The dataset analyzed during the current study is publicly available in the US Centers for Disease Control and Prevention 2010–2013 birth/infant death public files (https://www.cdc.gov/nchs/nvss/linked-birth.htm).

## Abbreviations

CDC: Centers for Disease Control and Prevention
CI: Confidence interval
OR: Odds ratio
